# Association between proteinuria and the development of malignant middle cerebral artery infarction: A retrospective cohort study

**DOI:** 10.1097/MD.0000000000030389

**Published:** 2022-09-16

**Authors:** Meng-Ni Wu, Pen-Tzu Fang, I-Hsiao Yang, Chung-Yao Hsu, Chiou-Lian Lai, Li-Min Liou

**Affiliations:** a Graduate Institute of Clinical Medicine, College of Medicine, Kaohsiung Medical University, Kaohsiung, Taiwan; b Department of Neurology, Kaohsiung Medical University Hospital, Kaohsiung, Taiwan; c Department of Radiation Oncology, Kaohsiung Medical University Hospital, Kaohsiung, Taiwan; d Department of Medical Imaging, Kaohsiung Medical University Hospital, Kaohsiung, Taiwan.

**Keywords:** brain edema, ischemic stroke, middle cerebral artery, proteinuria

## Abstract

A disrupted blood-brain barrier (BBB) with extravasation of macromolecules plays a critical role in the development of malignant middle cerebral artery infarction (MMI). Proteinuria is considered a marker of generalized endothelial dysfunction, including BBB disruption. This study aimed to clarify whether proteinuria identified in the acute stage of stroke is associated with MMI development. Patients with infarctions involving the middle cerebral artery territory were reviewed. Urine samples collected within 8 hours after stroke were analyzed using urine dipsticks. Patients were divided into proteinuria (urine dipstick reading of 1 + to 4+) and nonproteinuria groups. MMI was present if either signs of uncal herniation or a progressive conscious disturbance were recorded along with a midline shift > 5 mm identified on follow-up computed tomography (CT). Among the 1261 patients identified between January 2010 and June 2019, 138 were eligible for final analyses. Patients in the MMI group had lower Alberta Stroke Program Early CT Scores (ASPECTS), higher National Institutes of Health Stroke Scale scores, and a greater proportion of proteinuria than those in the non-MMI group. Four multivariate logistic regression models were used to clarify the role of proteinuria in MMI development. In model 1, proteinuria was significantly associated with MMI after adjusting for age, sex, dyslipidemia and ASPECTS (OR = 2.987, 95% CI = 1.329–6.716, *P* = .0081). The risk of developing MMI in patients with proteinuria remained significant in model 2 (OR = 3.066, 95% CI = 1.349–6.968, *P* = .0075) after adjusting for estimated glomerular filtrate rate (eGFR) < 60ml/min/1.73 m^2^ in addition to variables in model 1. In model 3, proteinuria was still significantly associated with MMI after adjusting for age, sex, dyslipidemia, ASPECTS, hypertension, diabetes, and atrial fibrillation (OR = 2.521, 95% CI = 1.075–5.912, *P* = .0335). In model 4, the risk of developing MMI in patients with proteinuria remained significant (OR = 2.579, 95% CI = 1.094–6.079, *P *= .0304) after adjusting for eGFR < 60ml/min/1.73 m^2^ in addition to variables in model 3. Proteinuria is independently associated with MMI development. Proteinuria may be a clinically accessible predictor of MMI development.

## 1. Introduction

Approximately 10% of infarctions involving the middle cerebral artery (MCA) territory may develop into malignant MCA infarction (MMI) with progressive disturbances in consciousness or signs of herniation combined with a shift in the midline structure.^[1]^ MMI may not only lead to profound disability and/or a vegetative state but may also contribute to a high mortality rate (up to 80%) if left untreated, which causes a tremendous socioeconomic burden.^[1,2]^ Early decompressive hemicraniectomy, within 48 hours after the onset of stroke, reduces mortality and severe disability.^[3,4]^ Therefore, early identification of patients at risk of MMI development is essential for providing timely therapy.

Studies on predictors of MMI have mainly focused on the analyses of demographic data, comorbidities, stroke severity, and imaging findings, including infarct volume, collateral circulation, or recanalization status.^[5–12]^ Analyses of these imaging findings are relatively technician-dependent and less clinically approachable. Additionally, few studies have investigated the predictors of MMI in terms of its pathophysiology. Pathologically, ischemia initiates cytotoxic and ionic edema. Subsequently, disruption of the blood-brain barrier (BBB) leads to extravasation of fluid, macromolecules, and blood cells, which contributes to vasogenic edema and hemorrhagic transformation.^[13,14]^ Protection of the BBB may potentially reduce vasogenic edema and hemorrhagic transformation after ischemic stroke.^[15]^ Severe leukoaraiosis and micro- and macroalbuminuria, as surrogates of preexisting disrupted BBB, have been reported to predict hemorrhagic transformation.^[16–18]^ Although a disrupted BBB also plays a critical role in MMI development,^[13,14]^ no study till date, to the best of our knowledge, has been designed to assess whether patients with preexisting disrupted BBBs are more vulnerable to extravasation than those with intact BBBs.

Proteinuria, a consequence of endothelial dysfunction in the kidney that allows hyperpermeability of macromolecules, is a predictor of stroke, coronary artery disease, and cardiovascular mortality.^[19–21]^ It is hypothesized that the kidney and brain share similar hemodynamic properties and common pathophysiologic processes related to risks.^[19,22,23]^ Therefore, proteinuria is considered a marker of generalized endothelial dysfunction, including glomerular dysfunction in impaired renal function and disrupted BBB integrity.^[19,22,24]^ In comparison to the evaluation of infarct volume, collateral circulation, and recanalization status, the assessment of proteinuria is less technician-dependent and more clinically approachable in routine practice. Therefore, this study aimed to clarify whether the presence of proteinuria in the acute stage of stroke, as a potential marker of a disrupted BBB, is associated with further development of MMI.

## 2. Methods

Patients with acute infarction involving the MCA territory who were admitted to the neuro-intensive care unit at Kaohsiung Medical University Hospital between January 2010 and June 2019 were retrospectively reviewed in this case-control study. The Institutional Review Board approved this study (KMUH-IRB-E(I)-20200147).

### 2.1. Participants

On admission, all patients underwent National Institutes of Health Stroke Scale (NIHSS) evaluation, brain computed tomography (CT), and laboratory tests, including urine analysis. After admission, patients underwent brain magnetic resonance imaging (MRI), including diffusion-weighted imaging (DWI). A follow-up brain CT was performed if any neurological deterioration was identified. Demographic characteristics, medical histories, and baseline laboratory reports were collected from patients’ medical records.

Patients were included in this study if they met the following criteria: (1) precisely established onset of symptoms; (2) DWI-confirmed infarction involving MCA territory with an Alberta Stroke Program Early CT Score (ASPECTS) of 7 or less; and (3) urine analysis using urine dipstick within 8 hours after stroke onset. One of the authors (M.N.W.) assessed the ASPECTS. intrarater reliability was represented by an intraclass correlation coefficient of 0.854.

Patients who met the following criteria were excluded: (1) any preexisting intracranial mass or encephalomalacia affecting the development of brain edema; (2) hemorrhagic transformation with either hemorrhagic infarction (HI)-2 with confluent petechiae in the infarcted zone, parenchymal hematoma (PH)-1 with hemorrhage involving <30% of infarcted zone and some mass effect, or PH-2 with hemorrhage involving >30% of infarcted zone or beyond the border of infarcted zone and some mass effect^[25]^; (3) receiving mechanical thrombectomy or intravenous thrombolysis; (4) involvement of other vascular territories; (5) evidence of urinary tract infection, including an increased white blood cell count and detectable bacteria or nitrites in the urine sample; (6) premorbid kidney diseases which could have caused proteinuria before infarction, including past histories of nephritic or nephrotic syndrome; (7) other conditions potentially masking neurological deterioration, including shock, sepsis, seizure, severe hypernatremia (serum sodium > 155 mg/dl) or hyponatremia (serum sodium < 125 mg/dl), or severe hypoglycemia (serum glucose < 60 mg/dl) within 5 days after infarction.

### 2.2. Potential risk factors

Hypertension and congestive heart failure were diagnosed based on the recorded history via medical chart review. Diabetes mellitus was determined based on the recorded history, use of oral antihyperglycemic agents or insulin, or a fasting sugar ≥ 126 mg/dL combined with a glycated hemoglobin > 6.5% identified after admission.^[26]^ Dyslipidemia was determined by a recorded history of dyslipidemia, use of cholesterol-lowering agents, total cholesterol > 160 mg/dL, or low-density lipoprotein cholesterol > 100 mg/dL.^[27,28]^ Atrial fibrillation was determined by chart review or any atrial fibrillation identified on 12-lead electrocardiogram or 24-hour Holter electrocardiogram results.

### 2.3. Proteinuria and estimated glomerular filtration rate

All patients underwent urine analysis and a survey of creatinine levels on admission. Using urine dipsticks, the urinary protein level was scored based on the dipstick reading as follows: negative (−), trace (+/−), 1+, 2+, 3+, and 4+. According to the urinary protein level, patients were divided into the proteinuria group (urine dipstick reading of 1 + to 4+) and the nonproteinuria group (urine dipstick reading that was negative or trace). The estimated glomerular filtration rate (eGFR) was calculated using the Modification of Diet in Renal Disease equation^[29]^:


$$\begin{array}{l} eGFR=186\times {\left(serum\;creatinine\right)}^{-1.154}\times age^{-0.203}\times 0.742\;\;\;\;\;\;\;(\text{female}) \end{array}$$

All patients were Asian. Patients were categorized into 2 groups: eGFR ≥ 60 and < 60 ml/min/1.73 m^2^.

### 2.4. Definition of malignant middle cerebral artery infarction

If any neurological deterioration was identified, a follow-up brain CT was performed. Patients were defined as having MMI if (1) either a progressive conscious disturbance (a decrease of consciousness ≥ 1 on item 1a of the NIHSS or a decline of total NIHSS ≥ 4) or signs of uncal herniation (a new onset of anisocoria with decreased light reflex or impaired ocular motility) were identified after admission; and (2) a midline shift > 5 mm at the level of the septum pellucidum was recorded in the follow-up brain CT images.^[9,30,31]^

### 2.5. Statistical analyses

Statistical analyses were conducted using the JMP software version 10 (SAS Institute, Cary, NC). Continuous variables are presented as mean ± standard deviation (SD) or median (interquartile range, IQR), as appropriate, and were analyzed using Student *t-test* (NIHSS) or the Mann–Whitney U test (age, eGFR, and ASPECTS), as indicated. Categorical variables are presented as numbers with proportions and were analyzed using the Chi-squared test. We used 4 multivariate logistic regression models to clarify the role of proteinuria in predicting MMI. In regression models 1 and 2, clinically meaningful variables (age and sex) and variables with a *P*-value < 0.1 in the univariate analysis (dyslipidemia and ASPECTS) were entered into the multivariate logistic regression analysis. In regression models 3 and 4, age, sex and variables with clinical significance (*P*-value < 0.2 in the univariate analysis), such as dyslipidemia, ASPECTS, hypertension, diabetes mellitus, and atrial fibrillation, were entered into multivariate logistic regression analysis. To clarify the impact of concurrent decreasing eGFR on the role of proteinuria in predicting MMI, models 2 and 4 were additionally adjusted for eGFR < 60 ml/min/1.73 m^2^ in combination with the variables already entered in models 1 and 3, respectively. There was no significant interaction between proteinuria and eGFR < 60 ml/min/1.73 m^2^ in both models 2 and 4 (Supplementary Tables S1 and S2, Supplemental Digital Content 1, http://links.lww.com/MD/H187). Statistical significance was set at *P* < .05.

## 3. Results

**Figure 1.**
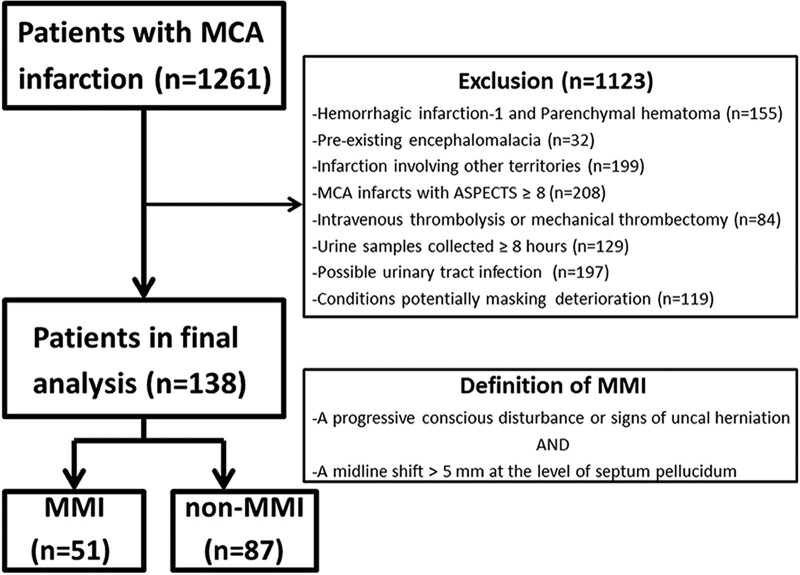
Algorithm for the identification and grouping of eligible. ASPECTS = Alberta Stroke Program Early Computed Tomography Score, MCA = middle cerebral artery, MMI = malignant middle cerebral artery infarction.

Among the 1261 patients with infarction involving the MCA territory, 1123 patients were excluded for the following reasons: 95 patients had HI-2; 60 had PH-1 or PH-2; 62 had involvement of the anterior cerebral artery territory; 137 had involvement of the brainstem, cerebellum or contralateral hemisphere; 208 had an infarction in the MCA territory with an ASPECTS of 8 or more; 84 were receiving intravenous thrombolytic therapy or mechanical thrombectomy; 32 had preexisting encephalomalacia; 129 did not have urine samples available within 8 hours after stroke onset; 197 had possible urinary tract infections; and, 119 had conditions potentially masking neurological deterioration (17 with shock, 51 with sepsis, 21 with hypernatremia, 18 with hyponatremia, 6 with seizure recorded clinically or identified by means of electroencephalogram, and 6 with severe hypoglycemia). The remaining 138 patients were eligible for further analyses. Figure 1 delineates the algorithm for the identification of eligible patients and their classification into the MMI and non-MMI groups.

Table 1 shows the demographic data, medical histories, ASPECTS, NIHSS scores on admission, presence of proteinuria, and eGFR for the MMI and non-MMI groups. The median age of the patients was 69 (62–80) years, and 73 (52.9%) patients were male. Fifty-one (37.0%) patients developed MMI. The interval between the onset of stroke and MRI was not significantly different between the groups (2.01 ± 0.78 days vs 1.82 ± 0.87 days, *P *= .1933). Patients in the MMI group had significantly higher NIHSS scores on admission (18.9 ± 5.2 vs 16.3 ± 5.1, *P *= .0055), lower ASPECTSs (2 [1–3] vs 4 [3–5], *P *< .0001), and a higher proportion of proteinuria (62.8% vs 32.2%, *P *= .0005) than those in the non-MMI group.

**Table 1:** Demographic characteristics in patients of MMI and non-MMI groups.

	Total (n = 138)	Non-MMI (n = 87)	MMI (n = 51)	*P*-value
Age, years, median (IQR)	69 (62–80)	72 (61–80)	68 (62–80)	0.6526
Sex (male), n (%)	73 (52.9)	46 (52.9)	27 (52.9)	0.9939
NIHSS, score, mean (±SD)	17.2 (5.3)	16.3 (5.1)	18.9 (5.2)	0.0055*
ASPECTS, score, median (IQR)	3 (2–5)	4 (3–5)	2 (1–3)	<0.0001*
Hypertension, n (%)	104 (75.4)	62 (71.3)	42 (82.4)	0.1445
Diabetes mellitus, n (%)	62 (44.9)	35 (40.2)	27 (52.9)	0.1473
Dyslipidemia, n (%)	84 (60.9)	58 (66.7)	26 (51.0)	0.0684
Congestive heart failure, n (%)	86 (62.3)	55 (63.2)	31 (60.8)	0.7758
Atrial fibrillation, n (%)	64 (46.4)	36 (41.4)	28 (54.9)	0.1241
Proteinuria, n (%)	60 (43.5)	28 (32.2)	32 (62.8)	0.0005*
eGFR (ml/min/1.73 m^2^), median (IQR)	75.2 (52.8–94.1)	76.0 (55.4–93.7)	71.6 (44.4–96.6)	0.5239

* *P* < .05.

Abbreviations: ASPECTS = Alberta Stroke Program Early Computed Tomography Score, eGFR = estimated glomerular filtration rate, MMI = malignant middle cerebral artery infarction, NIHSS = the National Institutes of Health Stroke Scale.

Sixty (43.5%) patients had proteinuria, and 48 (34.8%) patients had an eGFR < 60 ml/min/1.73 m^2^. There were 28 (20.3%) patients who had both proteinuria and eGFR < 60 ml/min/1.73 m^2^. Table 2 shows the demographic data, medical histories, ASPECTS and NIHSS scores on admission of patients in the proteinuria and nonproteinuria groups and of those in the eGFR < 60 and ≥ 60 ml/min/1.73 m^2^ groups. Patients in the proteinuria group and eGFR < 60 ml/min/1.73 m^2^ group had significantly higher NIHSS scores on admission, lower ASPECTSs, and higher proportions of hypertension, diabetes, and atrial fibrillation than those in the nonproteinuria group and the eGFR ≥ 60 ml/min/1.73 m^2^ group. A higher proportion of patients with proteinuria had eGFR < 60 ml/min/1.73 m^2^.

**Table 2:** Demographic characteristics of patients based on the proteinuria status (proteinuria and non-proteinuria) and the eGFR status (eGFR ≥ 60 and eGFR < 60).

	Proteinuria			eGFR		
	nonProteinuria (n = 78)	Proteinuria (n = 60)	*P*-value	eGFR ≥ 60 (n = 90)	eGFR < 60 (n = 48)	*P*-value
Age, years, median (IQR)	68 (59–80)	73 (63–81)	0.2373	68 (58–79)	75 (67–83)	0.0039*
Sex (male), n (%)	41 (52.6)	32 (53.3)	0.9285	50 (55.6)	23 (47.9)	0.3919
NIHSS, score, mean (±SD)	16.2 (5.3)	18.7 (5.0)	0.0057*	16.5 (5.2)	18.6 (5.1)	0.0319*
Hypertension, n (%)	52 (66.7)	52 (86.7)	0.0069*	63 (70.0)	41 (85.4)	0.0453*
Diabetes, n (%)	27 (34.6)	35 (58.3)	0.0055*	32 (35.6)	30 (62.5)	0.0024*
Dyslipidemia, n (%)	46 (59.0)	38 (63.3)	0.6030	55 (61.1)	29 (60.4)	0.9365
Heart failure, n (%)	43 (55.1)	43 (71.7)	0.0469*	52 (57.8)	34 (70.8)	0.1317
Atrial fibrillation, n (%)	28 (35.9)	36 (60.0)	0.0049*	31 (34.4)	33 (68.8)	0.0001*
ASPECTS, score, median (IQR)	4 (3–5)	3 (2–4)	0.0007*	4 (2–5)	3 (2–3)	0.0043*

* *P* < .05.

Abbreviations: ASPECTS = Alberta Stroke Program Early Computed Tomography Score, eGFR = estimated glomerular filtration rate, MMI = malignant middle cerebral artery infarction, NIHSS = the National Institutes of Health Stroke Scale.

Tables 3 and 4 summarize the univariate and multivariate logistic regression models, respectively. In the regression model 1, patients with proteinuria had a significantly higher risk of developing MMI (OR = 2.987, 95% CI = 1.329–6.716, *P *= .0081) than those without proteinuria after adjusting for age, sex, dyslipidemia and ASPECTS. In the regression model 2, the risk of developing MMI in patients with proteinuria remained significant (OR = 3.066, 95% CI = 1.349–6.968, *P* = .0075) after adjusting for eGFR < 60ml/min/1.73 m^2^ in addition to age, sex, dyslipidemia, and ASPECTS. In model 3, after adjusting for age, sex, dyslipidemia, ASPECTS, hypertension, diabetes mellitus, and atrial fibrillation, patients with proteinuria still had a significantly higher risk of developing MMI (OR = 2.521, 95% CI = 1.075–5.912, *P* = .0335) than those without proteinuria. In patients with proteinuria, the risk of developing MMI remained significant in model 4 (OR = 2.579, 95% CI = 1.094–6.079, *P *= .0304) after adjusting for eGFR < 60 ml/min/1.73 m^2^ in addition to age, sex, dyslipidemia, ASPECTS, hypertension, diabetes mellitus, and atrial fibrillation. The details of regression model 1–4 are illustrated in (Supplementary Tables S3–S6, Supplemental Digital Content 2, http://links.lww.com/MD/H188).

**Table 3:** The logistic regression analysis of MMI development to the proteinuria status without adjustment for estimated glomerular filtration rate.

	MMI (crude)			MMI (adjusted) (model 1)			MMI (adjusted) (model 3)		
	OR	95% CI	*P*-value	OR	95% CI	*P*-value	OR	95% CI	*P*-value
Proteinuria status									
Nonproteinuria	1.00			1.00			1.00		
Proteinuria	3.549	1.720–7322	0.0010*	2.987	1.329–6.716	0.0081*	2.521	1.075–5.912	0.0335*

* *P* < .05.

In the model 1, the regression was adjusted for age, sex, dyslipidemia, and ASPECTS with the independent variable set as proteinuria.

In the model 3, the regression was adjusted for age, sex, dyslipidemia, ASPECTS, hypertension, diabetes mellitus, and atrial fibrillation with the independent variable set as proteinuria.

Abbreviations: ASPECTS = Alberta Stroke Program Early Computed Tomography Score, MMI = malignant middle cerebral artery infarction.

**Table 4:** The logistic regression analysis of MMI development to the proteinuria status with adjustment for estimated glomerular filtration rate.

	MMI (adjusted) (model 2)			MMI (adjusted) (model 4)		
	OR	95% CI	*P*-value	OR	95% CI	*P*-value
Proteinuria status						
Nonproteinuria	1.00			1.00		
Proteinuria	3.066	1.349–6.968	0.0075*	2.579	1.094–6.079	0.0304*

* *P* < .05.

In the model 2, the regression was adjusted for renal function (estimated glomerular filtration rate < 60 ml/min/1.73 m^2^) in addition to age, sex, dyslipidemia, and ASPECTS. The independent variable was set as proteinuria.

In the model 4, the regression was adjusted for renal function (estimated glomerular filtration rate < 60 ml/min/1.73 m^2^) in addition to age, sex, dyslipidemia, ASPECTS, hypertension, diabetes mellitus, and atrial fibrillation. The independent variable was set as proteinuria.

Abbreviations: ASPECTS = Alberta Stroke Program Early Computed Tomography Score, MMI = malignant middle cerebral artery infarction.

## 4. Discussion

This study revealed that proteinuria identified within 8 hours after stroke onset is associated with an approximately 2- to 3-fold increased risk of developing MMI, independent of age, sex, dyslipidemia, hypertension, diabetes mellitus, or atrial fibrillation. Conversely, no significant relationship was found between the eGFR and the development of MMI. Furthermore, the impact of proteinuria on MMI development was independent of the eGFR.

Endothelial dysfunction with hyperpermeability to macromolecules underlies both glomerular protein leakage and disrupted BBB integrity.^[19,32]^ The Micro-vasculature in the brain and kidney may share similar hemodynamic and anatomical properties as well as common pathogenetic processes with similar risk factors, including diabetes, hypertension or hypercholesterolemia.^[19,22]^ The juxta-medullary afferent arterioles, the earliest and most severely injured anatomical site in patients with proteinuria, are directly exposed to high and fluctuating pulsatile pressure from large vessels, similar to the perforating arterioles of the brain.^[33,34]^ In a spontaneously hypertensive stroke-prone rat model, similar pathological vascular injuries were concurrently recorded in both perforating arterioles of the brain and juxtamedullary afferent arterioles.^[35]^ Therefore, proteinuria is considered a marker of generalized endothelial dysfunction, including disrupted BBB integrity.^[19,22]^ We postulated that patients with proteinuria in the acute stage of stroke may have concomitant disrupted BBBs which may contribute toward vulnerability to extravasation during vasogenic edema and a tendency of developing MMI.

Pathologically, a disrupted BBB with extravasation of intravascular content not only leads to MMI development, but also contributes to hemorrhagic transformation when blood cells, in addition to fluid and macromolecules, penetrate through this impaired mechanical and electrical barrier into the extravascular space.^[13,14]^ Proteinuria, considered a marker of a disrupted BBB, has been reported to be a predictor of hemorrhagic transformation after ischemic stroke in patients with and without reperfusion therapy.^[17,18]^ Despite the shared pathophysiology of MMI development and hemorrhagic transformation, to the best of our knowledge, no study, has assessed the association between proteinuria and MMI development.

Although the evaluation of BBB permeability by means of perfusion CT or Tc-99 m diethylenetriamine-pentaacetic-acid single-photon emission CT has been reported as a predictor of MMI development,^[36–38]^ these methods are relatively time-consuming and facility-dependent. Conversely, proteinuria assessment is relatively convenient, inexpensive and clinically accessible.

This study has some limitations. First, owing to the retrospective design, the impact of the severity and duration of diabetes mellitus, hypertension, and atrial fibrillation on MMI could not be clarified, and a causal relationship between proteinuria and the development of MMI could not be inferred. Second, acute stress-related microalbuminuria may have confounded the interpretation.^[39]^ However, a urine dipstick was used to measure macroalbuminuria, rather than microalbuminuria, to reduce the impact of stress-related microalbuminuria. A prospective cohort study should be conducted using parameters measured before stroke to confirm whether a premorbid BBB disruption can predict MMI development. Third, only patients at risk of developing MMI (ASPECTS ≤ 7) were included in this study, which may affect generalizability. Fourth, we could not precisely clarify recanalization status because of the retrospective design. Therefore, we excluded patients receiving intravenous thrombolysis or mechanical thrombectomy to diminish the impact of recanalization; this exclusion criterion may also affect generalizability.

## 5. Conclusions

In conclusion, this study revealed that proteinuria identified in the acute stage of stroke is associated with the further development of MMI. We speculate that proteinuria may be considered a clinically accessible and relevant predictor of MMI development. Further prospective cohort studies should be conducted to confirm whether a premorbid BBB disruption is vulnerable to extravasation during vasogenic edema and indicates a tendency to develop MMI.

## Acknowledgments

The authors thank the help from Dr Fu-Wen Liang, a biostatistician in the Division of Medical Statistics and Bioinformatics, Department of Medical Research, Kaohsiung Medical University Hospital, Kaohsiung Medical University.

## Author contributions

Conceptualization: Wu, M.N; Fang, P.T; Yang, I.H; Hsu, C.Y; Lai, C.L; Liou, L.M

Data curation: Wu, M.N; Fang, P.T; Yang, I.H; Liou, L.M

Formal analysis: Wu, M.N; Fang, P.T; Yang, I.H; Liou, L.M

Funding acquisition: Wu, M.N

Investigation: Wu, M.N; Fang, P.T; Yang, I.H; Liou, L.M

Methodology: Wu, M.N; Fang, P.T; Yang, I.H; Hsu, C.Y; Lai, C.L; Liou, L.M

Project administration: Wu, M.N; Liou, L.M

Resources: Wu, M.N; Fang, P.T; Yang, I.H; Liou, L.M

Software: Yang, I.H

Supervision: Hsu, C.Y; Lai, C.L; Liou, L.M

Validation: Wu, M.N; Liou, L.M

Visualization: Wu, M.N, Liou, L.M

Writing-original draft: Wu, M.N

Writing-review & editing: Wu, M.N; Hsu, C.Y; Lai, C.L; Liou, L.M

## Supplementary Material


