# Supplementary material for: Association between proteinuria and the development of malignant middle cerebral artery infarction: A retrospective cohort study
Source: Medicine (Baltimore). 2022 Sep 16;101(37):e30389. doi: 10.1097/MD.0000000000030389 (PMC9478230; doi:10.1097/MD.0000000000030389)
Supplement: Supplementary file 1 [file medi-101-e30389-s001.pdf]

S1 Table. The interaction between presence of proteinuria and eGFR in model 2

	OR	95% CI	*p*-value
Age, years	0.978	0.942 – 1.015	0.2416
Sex, male	0.854	0.362 – 2.014	0.7185
Dyslipidemia, yes	0.482	0.212 – 1.094	0.0810
ASPECTS, score	0.623	0.483 – 0.803	0.0003*
Proteinuria, yes	4.379	1.503 – 12.758	0.0068*
eGFR <60(ml/min/1.73 m^2), yes	1.424	0.383 – 5.298	0.5983
Proteinuria * eGFR <60	0.400	0.072 – 2.212	0.2936

* $p<0.05$

There was no significant interaction between eGFR < 60 ml/min/1.73 m^2 and proteinuria in model 2

Abbreviations: ASPECTS, Alberta Stroke Program Early Computed Tomography Score; eGFR, estimated glomerular filtration rate; MMI, malignant middle cerebral artery infarction

S2 Table. The interaction between presence of proteinuria and eGFR in model 4

	OR	95%CI	*p*-value
Age, years	0.974	0.936 – 1.014	0.1991
Sex, male	0.885	0.368 – 2.129	0.7854
Dyslipidemia, yes	0.501	0.217 – 1.158	0.1058
ASPECTS, score	0.613	0.472 – 0.797	0.0003*
Hypertension, yes	1.687	0.587 – 4.844	0.3313
Diabetes mellitus, yes	1.262	0.535 – 2.977	0.5949
Atrial fibrillation, yes	1.152	0.462 – 2.874	0.7610
Proteinuria, yes	3.545	1.154 – 10.889	0.0271*
eGFR <60(ml/min/1.73 m^2), yes	1.163	0.298 – 4.543	0.8281
Proteinuria * eGFR <60	0.459	0.081 – 2.609	0.3794

* $p<0.05$

There was no significant interaction between eGFR < 60 ml/min/1.73 m^2 and proteinuria in model 4

Abbreviations: ASPECTS, Alberta Stroke Program Early Computed Tomography Score; eGFR, estimated glomerular filtration rate; MMI, malignant middle cerebral artery infarction
