# Supplementary material for: Association between proteinuria and the development of malignant middle cerebral artery infarction: A retrospective cohort study
Source: Medicine (Baltimore). 2022 Sep 16;101(37):e30389. doi: 10.1097/MD.0000000000030389 (PMC9478230; doi:10.1097/MD.0000000000030389)
Supplement: Supplementary file 2 [file medi-101-e30389-s002.pdf]

S3 Table. The logistic regression of MMI development based on the presence of proteinuria after adjustment for age, sex, dyslipidemia and ASPECTS (Model 1)

	OR	95%CI	*p*-value
Age, years	0.980	0.946 – 1.016	0.2740
Sex, male	0.889	0.380 – 2.079	0.7852
Dyslipidemia, yes	0.506	0.225 – 1.139	0.1000
ASPECTS, score	0.629	0.492 – 0.805	0.0002*
Proteinuria, yes	2.987	1.329 – 6.716	0.0081*

* $p<0.05$

Abbreviations: ASPECTS, Alberta Stroke Program Early Computed Tomography Score; MMI, malignant middle cerebral artery infarction

S4 Table. The logistic regression of MMI development based on the presence of proteinuria after adjustment for age, sex, dyslipidemia, ASPECTS and eGFR (Model 2)

	OR	95% CI	*p*-value
Age, years	0.982	0.947 – 1.019	0.3333
Sex, male	0.892	0.381 – 2.088	0.7926
Dyslipidemia, yes	0.509	0.226 – 1.145	0.1024
ASPECTS, score	0.623	0.484 – 0.802	0.0002*
Proteinuria, yes	3.066	1.349 – 6.968	0.0075*
eGFR <60(ml/min/1.73 m^2), yes	0.829	0.347 – 1.983	0.6740

* $p<0.05$

Abbreviations: ASPECTS, Alberta Stroke Program Early Computed Tomography Score; eGFR, estimated glomerular filtration rate; MMI, malignant middle cerebral artery infarction

S5 Table. The logistic regression of MMI development based on the presence of proteinuria after adjustment for age, sex, dyslipidemia, ASPECTS, hypertension, diabetes and atrial fibrillation (Model 3)

	OR	95% CI	*p*-value
Age, years	0.976	0.939 – 1.014	0.2081
Sex, male	0.916	0.384 – 2.187	0.8442
Dyslipidemia, yes	0.525	0.230 – 1.198	0.1258
ASPECTS, score	0.621	0.481 – 0.803	0.0003*
Hypertension, yes	1.734	0.614 – 4.896	0.2985
Diabetes mellitus, yes	1.214	0.519 – 2.836	0.6550
Atrial fibrillation, yes	1.115	0.459 – 2.708	0.8099
Proteinuria, yes	2.521	1.075 – 5.912	0.0335*

* $p<0.05$

Abbreviations: ASPECTS, Alberta Stroke Program Early Computed Tomography Score; MMI, malignant middle cerebral artery infarction

S6 Table. The logistic regression of MMI development based on the presence of proteinuria after adjustment for age, sex, dyslipidemia, ASPECTS, hypertension, diabetes, atrial fibrillation and eGFR (Model 4)

	OR	95% CI	*p*-value
Age, years	0.978	0.940 – 1.016	0.2499
Sex, male	0.916	0.384 – 2.189	0.8441
Dyslipidemia, yes	0.531	0.233 – 1.211	0.1321
ASPECTS, score	0.613	0.472 – 0.796	0.0002*
Hypertension, yes	1.784	0.629 – 5.059	0.2764
Diabetes mellitus, yes	1.257	0.534 – 2.960	0.6000
Atrial fibrillation, yes	1.187	0.479 – 2.940	0.7110
Proteinuria, yes	2.579	1.094 – 6.079	0.0304*
eGFR <60(ml/min/1.73 m^2), yes	0.730	0.295 – 1.811	0.4977

* $p<0.05$

Abbreviations: ASPECTS, Alberta Stroke Program Early Computed Tomography Score; eGFR, estimated glomerular filtration rate; MMI, malignant middle cerebral artery infarction
